# Translating Population‐Level Trends of Aortic Dissection Mortality in Hypertension Into Clinical Insight

**DOI:** 10.1002/clc.70335

**Published:** 2026-04-24

**Authors:** Haruka Tanaka, Teruhiko Imamura

**Affiliations:** ^1^ Second Department of Internal Medicine University of Toyama Toyama Japan

**Keywords:** blood pressure, epidemiology, vessel disease

To the Editor,

We read with great interest the recent article, which examined temporal trends in mortality due to aortic dissection (AD) among adults with primary hypertension using the CDC WONDER database over two decades [1]. Several concerns have been raised.

The definition of “primary hypertension” based on death certificate coding (ICD‐10 I10) warrants careful consideration. Death certificate data are inherently limited by potential misclassification and underreporting, particularly for comorbid conditions [2]. As acknowledged by the authors [1], the absence of granular clinical data—including blood pressure levels, duration of hypertension, and treatment status—makes it challenging to determine whether hypertension is a causal contributor, a comorbidity, or a surrogate marker of overall cardiovascular risk burden. Additional sensitivity analyses—such as including broader hypertension‐related codes (e.g., I11–I13) or evaluating consistency across coding strategies—might strengthen confidence in the observed associations.

The interpretation of mortality trends requires caution. The reported pattern—an initial increase, followed by a decline and subsequent stabilization [1]—may reflect changes in diagnostic accuracy, imaging availability, and coding practices rather than true epidemiological shifts [3]. For instance, the widespread adoption of advanced imaging modalities, such as computed tomography angiography, during the study period may have improved detection of AD, particularly in earlier years. Conversely, temporal changes in hypertension awareness and control could confound the observed trends. Incorporating external data on hypertension control rates or healthcare access over time may help contextualize these findings.

The comparison between AD‐related mortality in hypertensive patients and the general AD population is intriguing but may be influenced by selection bias [1]. Since hypertension is highly prevalent in patients with AD, the distinction between these groups may not represent mutually exclusive populations. Clarification on how overlapping cases were handled, as well as proportional analyses might improve interpretability.

The lack of differentiation between Stanford type A and type B dissections represents a critical limitation [1]. These entities differ substantially in pathophysiology, therapeutic strategies, and clinical outcomes. Without this distinction, it is challenging to translate the findings into clinical decision‐making.

The observed geographic and racial disparities are important but remain largely descriptive. While the authors appropriately highlight higher mortality in non‐Hispanic Black populations and certain regions [1], the underlying mechanisms, including socioeconomic status, healthcare access, treatment delays, and comorbidity burden, are not directly assessed. Multivariable or mediation analyses incorporating area‐level socioeconomic indicators could help disentangle these complex relationships [4].

## Funding

The authors have nothing to report.

## Ethics Statement

The authors have nothing to report.

## Consent

The authors have nothing to report.

## Conflicts of Interest

The authors declare no conflicts of interest.

## Data Availability

The manuscript does not include any original data.
